# Dengue virus and its recent outbreaks: current scenario and counteracting strategies

**DOI:** 10.1097/JS9.0000000000000045

**Published:** 2023-03-01

**Authors:** Ranjit Sah, Abdelmonem Siddiq, Bijaya K. Padhi, Aroop Mohanty, Ali A. Rabaan, Deepak Chandran, Chiranjib Chakraborty, Kuldeep Dhama

**Affiliations:** a Department of Microbiology, Tribhuvan University Teaching Hospital, Institute of Medicine, Kathmandu, Nepal; b Department of Microbiology, Dr. D. Y. Patil Medical College, Hospital and Research Centre, Dr. D. Y. Patil Vidyapeeth, Pune, Maharashtra; c Datta Meghe Institute of Higher Education and Research, Jawaharlal Nehru Medical College, Wardha, India; d Faculty of Pharmacy, Mansoura University, Mansoura, Egypt; e Department of Community Medicine and School of Public Health, Postgraduate Institute of Medical Education and Research, Chandigarh; f All India Institute of Medical Sciences, Gorakhpur, India; g Molecular Diagnostic Laboratory, Johns Hopkins Aramco Healthcare, Dhahran; h College of Medicine, Alfaisal University, Riyadh, Saudi Arabia; i Department of Veterinary Sciences and Animal Husbandry, Amrita School of Agricultural Sciences, Amrita Vishwa Vidyapeetham University, Coimbatore, Tamil Nadu; j Department of Biotechnology, School of Life Science and Biotechnology, Adamas University, Kolkata, West Bengal; k Division of Pathology, ICAR-Indian Veterinary Research Institute, Bareilly, Izatnagar, Uttar Pradesh, India

*Dear Editor*,

Dengue, or breakbone fever, is a viral infection which is considered the most rapidly transmitted vector-borne disease to humans by the *Aedes aegypti* female mosquito and places a significant strain on global public health services. In addition to underreporting, many dengue cases are also misclassified. Mortality and morbidity continue to be big issues while we remain in the era of supportive care^1^. The WHO has designated dengue as a serious international public health issue because of the viruses and mosquitoes increasing global distribution, the recurrence of epidemics occasion, and the emergence of dengue fever in new regions^1^. More than 100 nations have reported cases of endemic dengue fever, and the annual number of new cases is estimated to be around 390 million. These numbers have been rising steadily, as reported by the WHO^2^. The fatality rate for severe dengue, which accounts for 3–4% of all dengue fever infections, is 2–5% when properly treated but can reach 50% if untreated. Co-infection with other microbes increases the risk of severe sickness and death in dengue patients^3^. Dengue fever is an infection caused by one of the four dengue serotypes (dengue virus [DENV]-1, DENV-2, DENV-3, and DENV-4). The virus attaches to the cell surface and is taken inside via endocytosis, where it is encased in an endosome and exposed to the acidic environment necessary for its replication. The virus then uses the rough endoplasmic reticulum and the ribosomes to produce a polypeptide chain consisting of 10 proteins, three of which are structural and seven of which are nonstructural. Although the virus’s four serotypes share a genetic similarity of just about 65%, they all manifest clinically in the same ways^4^. Dengue virus subtype infections can be asymptomatic or cause fever with varying degrees of severity, from mild flu-like symptoms to life-threatening states like dengue hemorrhagic fever and dengue shock syndrome^5^.

Dengue fever is spread through the bite of an infected mosquito, specifically the *Ae. aegypti* mosquito; other species of the same genus are also known to transmit the disease in tropical and subtropical regions. Human skin immature dendritic cells are the initial target, with the infection spreading to nearby lymph nodes, the liver, the spleen, and even monocytes in the bloodstream. The virus can also be passed from an infected mother to her unborn child through the placenta or during delivery^6,7^.

Dengue fever has spread to more than a hundred nations, making it the most common arbovirus worldwide. It has now reached different regions of the WHO, such as the Americas, Africa, the Eastern Mediterranean, Southeast Asia, and the Western Pacific, with the Americas, Southeast Asia, and the Western Pacific being the hardest hit. WHO received reports of 505.430 million cases of dengue fever in 2000, 2.4 million in 2010, and 502 million in 2019, with fatalities rising from 960 in 2000 to 4032 in 2015. The first dengue cases were reported in France and Croatia in 2010; in 2012, there was an outbreak that resulted in 2000 cases; and in 2019, all regions are afflicted by a massive outbreak, with the Americas reporting 3.1 million cases, of which 25 000 were severe and a few resulted in death^8^.

Figure 1 shows, using data adapted from the WHO, the distribution of dengue fever cases among the five WHO regions from 1990 to 2017, together with the number of fatalities, the fatality rate, the incidence, and the expected number of new cases every day^9^.

**Figure 1:**
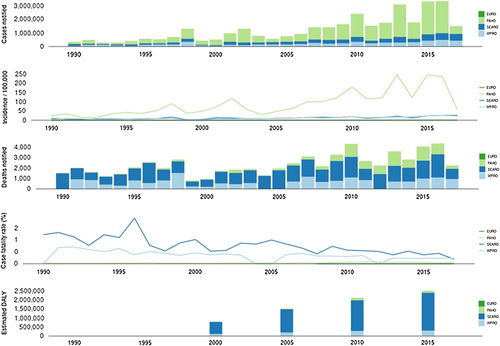
Describe the distribution of the dengue fever over the four regions of WHO including incidence, death rate, case fatality, and estimated daily cases^9^.

Figure 2 shows the current global situation with respect to dengue fever can be broken down into three groups: (1) nations where the virus appears consistently; (2) countries with occasional instances; and (3) countries where there is no evidence of the disease risk^10^.

**Figure 2:**
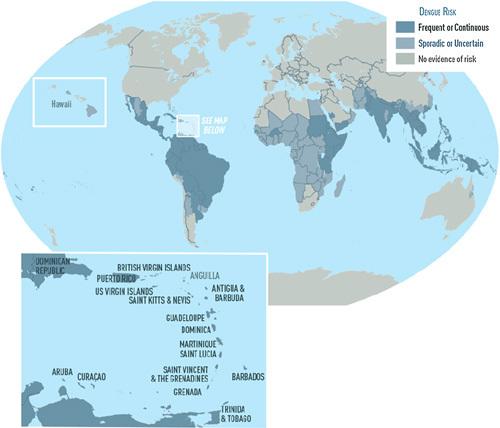
Describing the disease occurrence in different countries over the world as divided into continuous, sporadic, and no risk evidence^10^.

The estimated cases and deaths of the disease in individual countries are adopted from the dengue Map: Vietnam (77 000), Philippines (65 000), Laos (17 892), Ho Chi Minh, Vietnam (16 057), India (10 127), Honduras (7263), Karachi, Sindh, Pakistan (1729), Bangladesh (1056) with the reported death cases as follows: 274 cases in the Philippines, five cases in Negros Occidental, Philippines, one case in Bangladesh, Iloilo, Philippines, and one case in Dhaka, Bangladesh^11,12^.

The reported cases of dengue fever between June and August 2022 are presented in Fig. 3. Regarding the European Center for Disease Prevention and Control (ECDC) the last update on August 24, 2022, 2 597 067 total reported cases were as follow, Brazil (1 910 657), Vietnam (145 536), Philippines (82 597), Indonesia (68 903), and Peru (57 469) and the total death cases of 2067 as follows: Brazil (774), Vietnam (53), Philippines (319), Indonesia (640), and Peru (72)^13^.

**Figure 3:**
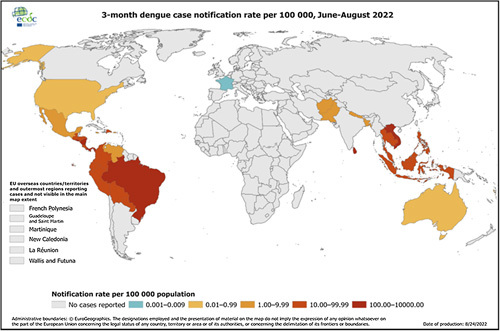
Describing the cases of dengue fever rate in the period from June to August to identify the countries where most commonly occur and others where no cases were reported^13^.

Dengue cases are being reported in epidemic proportions from neighboring Asian countries like Nepal, India, and Bangladesh. In Nepal, the top 10 districts have reported the highest number of cases, that is, 84% of total cases reported from 75 districts. Thirty-two deaths have been documented so far due to dengue in Nepal this year. For the response to dengue, the Epidemiology and Disease Control Division of Nepal has conducted stakeholder meetings for dengue prevention during the rainy season in rural and urban localities^14^.

In India, the national capital city of Delhi is experiencing an alarming spike in the number of dengue cases; however, no fatalities have been reported so far. The total number of cases in the previous 2 months has been reported to be 400; 75 of them were reported in August. The dengue cases are said to be reported usually between July and November months in India. On the other hand, Mumbai, the financial capital also witnessed a significant spike in dengue cases in the month of August. Cases are also being recorded in other states of the country, like Andhra Pradesh, Tamil Nadu, Telangana, Karnataka, Maharashtra, West Bengal, and Uttarakhand. According to official data released by the National Vector Borne Disease Control Program (NVBDCP), as of August 30, 2022, India had logged 30 627 total dengue cases and 12 deaths. Comparatively, in the previous year 2021, the total number of cases was one, 93 245 with 36 deaths. The trend shows a continuing spike in the number of cases with on-going weather conditions^15^. Bangladesh logged 392 dengue hospitalizations in 1 day in September 2022. The total number of dengue hospitalizations reached 1483. So far, this year has recorded 11 569 dengue cases and a total of 45 causalities. The maximum number of cases is coming from the capital city Dhaka^16^.

The laboratory detection of the virus can be done through different methods, including:

1. Virus isolation: the isolation of the virus from its natural host, whether that be a patient’s serum during the viremia phase, plasma, leukocytes, blood, or tissues, or even during the viral replication phase using certain techniques like the PCR, is widely regarded as the most specific method^17^.
2. Serological testing: to detect high immunoglobulin G levels from a previous infection or high immunoglobulin M levels from the current acute infection, which are present after 1 week of the infection until about 3 months later using the enzyme-linked immunosorbent assay, serological testing is the most commonly used method because it is inexpensive and relatively available compared to other techniques of viral isolation or nucleic acid detection^18^.
3. Nucleic acid amplification: methods for dengue virus detection using nucleic acid amplification, include reverse transcriptase PCR, real-time PCR, and nucleic acid sequence-based amplification tests but these are not yet commercially accessible^19^.
4. Viruses can be detected through the detection of antigens such as the NS1 antigen, which is a glycoprotein generated by all flaviviruses because of its importance for the replication and vitality of the virus and secreted in the bloodstream.

As there is no specific treatment recommended for dengue, supportive therapy is the mainstay of treatment. This includes fluid replacement therapy, such as 0.9% saline as a first line, which can be given as an infusion or as a bolus dose in case of shock; this is supported by evidence to prevent hypovolemia and the occurrence of complications. While acetaminophen can be used as an antipyretic, NSAIDs should be prohibited as they increase the risk of bleeding. The patient’s situation will dictate whether or not they require hospitalization, so they should be evaluated and monitored closely for any signs of trouble^1^.

Currently, only Denvaxia (Sanofi Pasteur Inc. USA) has been granted licensing, but TV-003/TV-005 and TAK-003 are undergoing promising phase III clinical trials^20^. Dengvaxia is approved for use in children aged 9–16 years who had experienced a previous dengue infection confirmed virologically or in people living in endemic areas of the disease; however, this vaccine is not recommended for the patient who does not have a history of dengue infection because it has been shown that these people will subsequently become infected with dengue after receiving the vaccine.

Vector control, including the use of larvicide and adulticide, the prevention of mosquito breeding by covering standing water, the use of chemical agents containing active substances like N,N-Diethyl-meta-toluamide and picaridin to keep water safe, the use of insect repellent, the use of long clothing treated with permethrin as an insecticide, and other measures, is essential for the prevention of dengue fever. Covering windows and implementing measures to prevent the entrance of mosquitos, such as mosquito nets, providing educational programs about the disease transmission and preventative measures on how to protect themselves from the disease to increase the awareness of the general population and make them cooperative with the public health specialists, implementing a surveillance program to identify the prevalence of the disease so that we can tailor our efforts to hinder the spread, and promoting the scientific research in this area to identify more safe insecticides are effective techniques to stop the outbreak of dengue fever.

Large portions of the world’s population are in danger as dengue fever spreads throughout the tropics and beyond, as the human and environmental prerequisites for its persistence and even spread are present on all continents. Dengue hemorrhagic fever and its sequelae cause a substantial number of deaths, and the disease is extremely expensive as well as incredibly painful for those affected. This problem has been worse recently, and it may get worse again. There has been no success in reducing transmission by vector management, and there is currently no antiviral medication that is either widely available or on the horizon that could significantly reduce transmission. As can be seen from the available clinical data on dengue vaccines, the development of an effective and safe vaccine that provides protection against all four serotypes of the virus without triggering any immunopathogenic events in subsequent DENV encounters is a unique challenge. It is mandatory to greatly improve vector control measures in the endemic areas of dengue and promote research for the development of a universal and effective vaccine to break the dengue transmission chain, as the dengue virus’s emergence in a new region of the world as an epidemic disease has been potentiated in number and occurrence despite all efforts. As long as safety concerns persist, getting a dengue vaccine that works and is safe to use remains an urgent priority.

## Ethical approval

None.

## Sources of funding

None.

## Author contribution

R.S. and A.S.: draw and design the draft; B.K.P., A.M., A.A.R., D.C., C.C. and K.D.: review the literature and critically edit the manuscript. All authors read and agrees for the final manuscript.

## Conflicts of interest disclosure

The authors declare that they have no financial conflict of interest with regard to the content of this report.

## Research registration unique identifying number (UIN)

None.

## Guarantor

Ranjit Sah.
